# Time–Frequency Analysis of Particulate Matter (PM_10_) Concentration in Dry Bulk Ports Using the Hilbert–Huang Transform

**DOI:** 10.3390/ijerph17165754

**Published:** 2020-08-09

**Authors:** Xuejun Feng, Jinxing Shen, Haoming Yang, Kang Wang, Qiming Wang, Zhongguo Zhou

**Affiliations:** 1 College of Habour, Coastal and Offshore Engineering, Hohai University, No.1, Xikang Road, Nanjing 210098, China; fxj@hhu.edu.cn (X.F.); wk2019@hhu.edu.cn (K.W.); 2 College of Civil and Transportation Engineering, Hohai University, No.1, Xikang Road, Nanjing 210098, China; 3 Jiangsu Key Laboratory of Atmospheric Environment Monitoring and Pollution Control, Nanjing University of Information Science and Technology, No.219, Ningliu Road, Nanjing 210044, China; yanghm@jshb.gov.cn; 4 College of Science, Hohai University, No.1, Xikang Road, Nanjing 210098, China; wqm@hhu.edu.cn (Q.W.); zhgzhou@hhu.edu.cn (Z.Z.)

**Keywords:** ambient particulate matter, Hilbert–Huang transform (HHT), empirical mode decomposition (EMD), intrinsic mode functions (IMF), Hilbert spectrum analysis (HSA)

## Abstract

To analyze the time–frequency characteristics of the particulate matter (PM_10_) concentration, data series measured at dry bulk ports were used to determine the contribution of various factors during different periods to the PM_10_ concentration level so as to support the formulation of air quality improvement plans around port areas. In this study, the Hilbert–Huang transform (HHT) method was used to analyze the time–frequency characteristics of the PM_10_ concentration data series measured at three different sites at the Xinglong Port of Zhenjiang, China, over three months. The HHT method consists of two main stages, namely, empirical mode decomposition (EMD) and Hilbert spectrum analysis (HSA), where the EMD technique is used to pre-process the HSA in order to determine the intrinsic mode function (IMF) components of the raw data series. The results show that the periods of the IMF components exhibit significant differences, and the short-period IMF component provides a modest contribution to all IMF components. Using HSA technology for these IMF components, we discovered that the variations in the amplitude of the PM_10_ concentration over time and frequency are discrete, and the range of this variation is mainly concentrated in the low-frequency band. We inferred that long-term influencing factors determine the PM_10_ concentration level in the port, and short-term influencing factors determine the difference in concentration data at different sites. Therefore, when formulating PM_10_ emission mitigation strategies, targeted measures must be implemented according to the period of the different influencing factors. The results of this study can help guide recommendations for port authorities when formulating the optimal layout of measurement devices.

## 1. Introduction

Dry bulk ports play an important role in promoting the development of global trade and economic integration [1]. According to the data released by the United Nations Conference on Trade and Development (UNCTAD) 2018, dry bulk cargoes, represented by iron ore, grains, and coal, account for about 53.3% of the global seaborne trade volumes that are handled by ports [2]. However, the particulate matter (PM) emitted by dry bulk cargoes during loading, storage, and transportation activities increase the ambient PM concentrations in the port and surrounding areas, which significantly negatively impacts public health [3,4,5,6,7,8]. Notably, particles with aerodynamic diameters less than 10 μm require particular consideration [9,10], as they can directly enter the lungs or even the bloodstream, so long-term exposure to such particles can cause severe cardiopulmonary diseases and even lung cancer [11,12,13,14,15]. Therefore, the variation in PM_10_ concentrations must be studied in dry bulk ports, especially on a range of temporal and spatial scales, so that supervisors can design targeted mitigation measures to reduce the risk of worker exposure to high concentrations of PM_10_.

To date, studies of PM_10_ concentrations in ports have mainly concentrated on quantifying the impact on the ambient air quality [16,17,18]. By comparing the average value of the PM_10_ concentration measured in the ports within a specific time frame, some researchers have discovered that the PM_10_ concentration level has distinct seasonal variation characteristics; however, the variation is not uniform in different regions [19,20]. Studies have shown that through the analysis of the chemical composition of the particulate matter combined with source analysis technology, the contribution of the port to the PM_10_ concentration of the surrounding cities could be accurately determined [21,22]. However, these commonly used statistical methods cannot provide more details about the differences in the PM_10_ concentration data in the time domain. In dry bulk ports, because of the combined impact of loading and unloading activities, terrain, wind speed, temperature, humidity, and the location of detectors, the fluctuation in the PM_10_ concentration data measured within a particularized period is complex. Given the combined influence of multiple factors, the fluctuation is non-linear and non-stationary. Therefore, an essential issue that needs to be urgently solved is how to conduct in-depth analyses of these particular time-series data in order to determine the periodicity and randomness of the components influencing the PM_10_ concentration levels on various time scales.

Our primary objectives in this study were to investigate the temporal characteristics of the PM_10_ concentration levels in a dry bulk port. With the PM_10_ concentration data measured at three different locations in the Xinglong port area of Zhenjiang, China, we mainly focused on (1) analyzing the PM_10_ concentration data and interpreting the potential fluctuation characteristics in the time domain, and (2) identifying the factors that influence the time–frequency characteristics of the PM_10_ concentration data series at different measurements to encourage port authorities to formulate targeted PM pollution control policies. The remainder of this paper is structured as follows: In Section 2, the data measurement equipment, period, and measurement position used in this study are introduced. In Section 3, the non-parametric statistical methods and the HHT employed in this study are described. Section 4 introduces and discusses the research results. Finally, the last section presents the main conclusions.

## 2. Materials

### 2.1. Area Description

**Figure 1:**
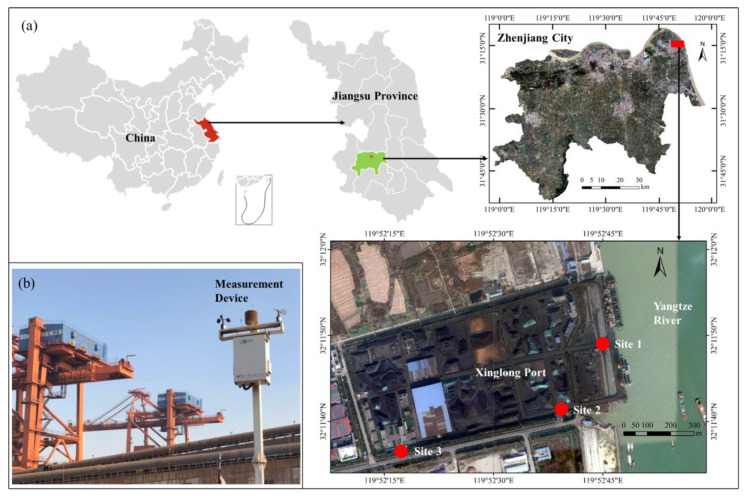
Study area and measurement device: (**a**) location of the city of Zhenjiang and the port of Xinglong; (**b**) installation of the measurement device.

The study was conducted at Xinglong Port in the east of Zhenjiang, which is a famous port city in the Yangtze River basin, located in the south of Jiangsu, Eastern China (Figure 1a). Xinglong Port, located on the south bank of the lower course of the Yangtze River, is one of the essential dry bulk ports in Zhenjiang, occupying a length of about 470 m along the shoreline of the Yangtze River. The port currently has one grocery berth of 30,000 deadweight tonnage and one bulk cargo berth of 35,000 deadweight tonnage, mainly providing service functions, including the loading, trading, and warehousing of iron ore, coal, phosphate, etc. The port yard area is about 210,000 m^2^, and the average annual throughput is about 5,000,000 tons.

In June 2019, to monitor the air quality of Xinglong Port according to the requirements of the Department of Ecology and Environment of Jiangsu Province, three measurement sites were selected in the port in order to measure the concentration of PM_10_ in the ambient air. As shown in Figure 1a, Site 1 (32°11′49.30″ N, 119°52′46.15″ E) was located on the north side of the port, near the berths where the unloading operations occur; Site 2 (32°11′41.90″ N, 119°52′39.30″ E) was situated on the east side of the port yard, where cargo is transported by trucks to other destinations; and Site 3 (32°11′36.90″ N, 119°52′17.10″ E) was on the outermost side of the port yard.

### 2.2. Measurements

The measurement devices were set to the height of 3.5 m above the ground, as shown in Figure 1b, so as to measure the PM_10_ and meteorological parameters, such as humidity, temperature, wind speed, and wind direction, in one-minute intervals, which satisfies the requirements of China Standard HJ 655-2013 (http://www.cnemc.cn/jcgf/dqhj/). The particles entered the devices at a flow rate of 16.7 L/min through an air sampler pump supplying a constant volumetric flow rate. In this study, the sensor used for PM_10_ measurement (Nova SDS011, FCC certification, Nova Fitness Co., Ltd., Jinan International Innovative Design Industrial Park, No2, Wanshou Road, Jinan, Shandong Province, China) uses the principle of laser scattering to measure PM_10_ concentrations between 0 and 999.9 µg/m^3^ with an accuracy of ± 10%. The humidity and temperature sensor (Digital Humidity Sensor SHT85, CE certification, Sensirion AG, Laubisruetistrasse 50, 8712 Staefa ZH, Switzerland) can measure the relative humidity between 0% and 100% with an accuracy of ± 1.5%, and measure temperature between −40 and 105 °C with an accuracy of ± 0.1 °C.

The port authorities completed the installation of the measuring devices in August 2019. The environmental supervisor commissioned and calibrated the devices in September 2019, and they checked the devices once a month to ensure the quality of the PM_10_ concentration values. Considering the impact of novel coronavirus 2019 COVID-19 on port shipping activities in 2020, the period of PM_10_ concentration data in this study ranged from 1 October to 30 December 2019. When the environmental supervisor checked the measurement device once per month, the power supply to the equipment needed to be stopped, which caused the loss of measurement data. Therefore, during the study, a total of 390,600 PM_10_ concentration values were obtained from these three measurement sites, and 2520 values were lost during the device detection process. In line with previous studies [23,24], the data were further analyzed after averaging to the hourly means, and about 2016 PM_10_ concentration values were measured at each site after removing the incomplete data.

## 3. Methodology

The Hilbert–Huang transform (HHT) was proven to be an effective method for analyzing non-linear and non-stationary data, and is particularly effective for decomposing complex time series data and obtaining intrinsic modal functions with specific cycles [25]. By decomposing the complex time series data into intrinsic mode functions (IMFs) with trend characteristics and estimating the amplitude and frequency of IMFs, HHT can be used to obtain more information about the features of the time series fluctuations than basic statistics such as the mean, standard deviation, and skewness [26]. Because of the advantages of HHT in analyzing non-stationary and non-linear time series data, this method is widely used in various professional fields, such as geophysics, health monitoring, ocean engineering, chemical engineering, and financial analysis [27,28,29,30,31,32,33]. HHT has also been thoroughly applied in atmospheric turbulence and meteorological analyses [34,35].

The HHT generally comprises two sequential steps, namely: empirical mode decomposition (EMD) and Hilbert spectrum analysis (HSA). The EMD methods can decompose the time-series data into eigenmode intrinsic mode functions (IMFs) with trends, and then the instantaneous frequency data can be obtained using the HSA technique [26].

### 3.1. Empirical Mode Decomposition

EMD can decompose the PM_10_ concentration data, x(t), measured within a specified time-lapse, into the sum of a series of intrinsic mode functions (IMFs), $c_{i}(t)$, and a residual term, $r_{n}(t)$, as follows:

(1) $$x(t)=\sum_{i=1}^{n}c_{i}(t)+r_{n}(t)$$

where IMF, $c_{i}(t)$, needs to satisfy the fundamental condition that the mean value is zero, and the total number of extreme points and the number of zero-crossing points must be equal or not differ by more than one. Each IMF is a simple harmonic function, and the time lapse between consecutive extreme values was selected as the time scale of the intrinsic oscillatory mode, which can provide an oscillation mode with an excellent resolution. Generally, the extraction of the IMF is a continued iterative sifting procedure. First, we determined all of the local maxima and local minima of data series, x(t), and generated the upper envelope $e_{u}(t,1)$ and lower envelope $e_{l}(t,1)$ by applying cubic splines in order to connect all of the local maxima and local minima, separately. The mean of those two envelopes, m(t,1), can be calculated using the following:

(2) $$m(t,1)=\frac{e_{u}(t,1)+e_{l}(t,1)}{2}$$

Then, the first component, h(t,1), can be extracted by the difference between the data series of x(t) and m(t,1), as shown in

(3) h(t,1)=x(t)−m(t,1)

If h(t,1) satisfies the fundamental conditions for IMF, then designate h(t,1) as $c_{1}(t)$; otherwise, replace x(t) with m(t,1), and repeat Equations (2) and (3) until h(t,k) satisfies the fundamental conditions for IMF. Under this condition, h(t,k) is designated as $c_{1}(t)$. Finally, replace x(t) with $x(t)-c_{1}(t)$, and repeat the above procedure to obtain $c_{2}(t),c_{3}(t),\ldots ,c_{n}(t)$ until the residue $r_{n}(t)$ is small enough or no further IMFs can be extracted.

### 3.2. Hilbert Spectrum Analysis

The corresponding instantaneous frequencies can be obtained through applying the Hilbert transform to the IMF components of the PM_10_ concentration data. The Hilbert transform for any IMF component $c_{i}(t)$ can be expressed as $H(c_{i}(t))$ in the following:

(4) $$H(c_{i}(t))=\frac{1}{\pi }P\int_{-\infty }^{\infty }\frac{c_{i}(\varphi )}{t-\varphi }d\varphi$$

where p is a constant that indicates the Cauchy principal value. In this condition, $c_{i}(t)$ and $H(c_{i}(t))$ form the complex conjugate pair, and the analytic data series $Z(c_{i}(t))$ of $c_{i}(t)$ can be defined by

(5) $$\left\{ \begin{array}{l} Z(c_{i}(t))=c_{i}(t)+jH(c_{i}(t))=a_{i}(t)e{}^{j\theta_{i}(t)}\\ a_{i}(t)=\sqrt{\left[H{\left(c_{i}(t)\right)}^{2}+c_{i}{\left(t\right)}^{2}\right]}\\ \theta_{i}(t)=\arctan(\frac{H(c_{i}(t))}{c_{i}(t)}) \end{array}\right.$$

where j is the fictitious component of $Z(c_{i}(t))$, $a_{i}(t)$ is the time-varying amplitude function, $\theta_{i}(t)$ is the time-varying phase angle function, and the instantaneous frequency of $Z(c_{i}(t))$ can be expressed as $\omega_{i}(t)$ in the following:

(6) $$\omega_{i}(t)=\frac{d\theta_{i}(t)}{dt}$$

Now, the Hilbert spectrum of the IMF component, $c_{i}(t)$, can be defined as $H(\omega_{i}(t),t)$ in Equation (7), and the original PM_10_ concentration data series x(t) can be represented as follows:

(7) $$H(\omega_{i}(t),t)=a_{i}(t),\forall \omega_{i}(t)$$

(8) $$x(t)=\sum_{i=1}^{n}a_{i}(t)e^{j\int \omega_{i}(t)dt}$$

Thus, from the Hilbert spectrum, $H(\omega_{i}(t),t)$, the instantaneous energy, $E_{i}(t)$, can be calculated using Equation (9), and the marginal spectrum $M_{i}(t)$ can be calculated by Equation (10):

(9) $$E_{i}(t)=\int_{\omega }H^{2}(\omega_{i}(t),t)d\omega$$

(10) $$M_{i}(t)=\int_{t}H^{2}(\omega_{i}(t),t)dt$$

## 4. Results and Discussion

### 4.1. Overview of PM_10_ Concentration Data

**Figure 2:**
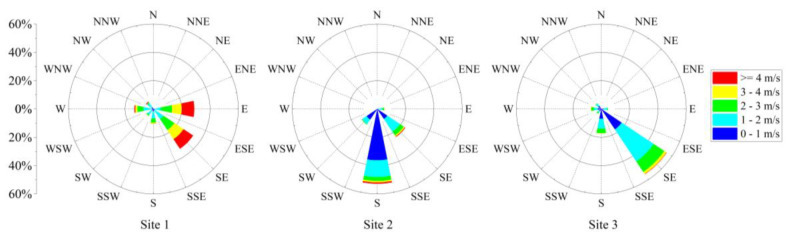
Wind rose for the three measurement sites over a three-month period.

Using the wind speed and wind direction data obtained from the three measurement sites in Xinglong Port, the generated wind rose plots are shown in Figure 2.

We observed that the average wind speed of Site 1 was 2.7 m/s, and the wind was blowing predominantly from the east (E) and southeast (SE) sectors. During the sampling period, the E and SE wind directions accounted for approximately 28.9% and 33.8% of the total wind, respectively. At Site 2, the average wind speed was 1.0 m/s, and the prevailing wind directions were SE and south (S), accounting for approximately 23.8% and 52.9%, respectively. At Site 3, the average wind speed was 1.3 m/s, SE was the prevailing wind direction with a non-negligible contribution from the S, accounting for approximately 55.5% and 17.4%, respectively. The main reason for the diversity in wind speed and direction at different measurement sites within the port may be the installation of a windbreak fence and other dust suppression measures around the harbor [36].

**Figure 3:**
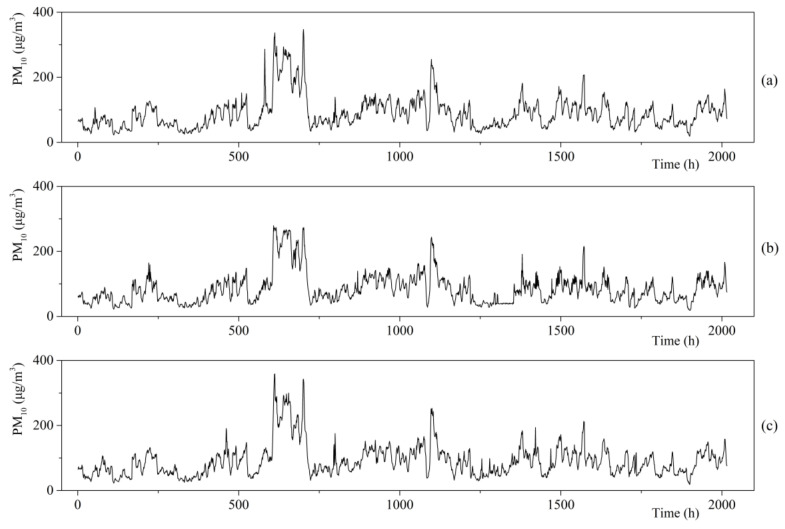
Time-varying characteristics of hourly particulate matter (PM_10_) concentration: (**a**) the data measured at Site 1; (**b**) the data measured at Site 2; (**c**) the data measured at Site 3.

Similarly, using the PM_10_ concentration values measured at the three different sites in Xinglong Port, the waveform of the data series was obtained, as shown in Figure 3.

The statistical characteristics of the PM_10_ concentration data are shown in Table 1.

Table 1 shows that the maximum values of the average PM_10_ concentration per hour at Sites 1, 2, and 3 reached 346.9, 279.11, and 358.77 μg/m^3^, respectively, and the mean values of the average PM_10_ concentration per hour at Sites 1, 2, and 3 were 88.77, 84.61, and 90.91 μg/m^3^, respectively. The average hourly data of the PM_10_ concentration indicates that a specific difference existed in the PM_10_ levels measured at these three sites. To further determine the difference in the PM_10_ concentration values obtained from different measurement sites, we applied EMD and HSA to investigate the time–frequency characteristics of the data.

### 4.2. EMD of the PM_10_ Concentration Data Series

**Figure 4:**
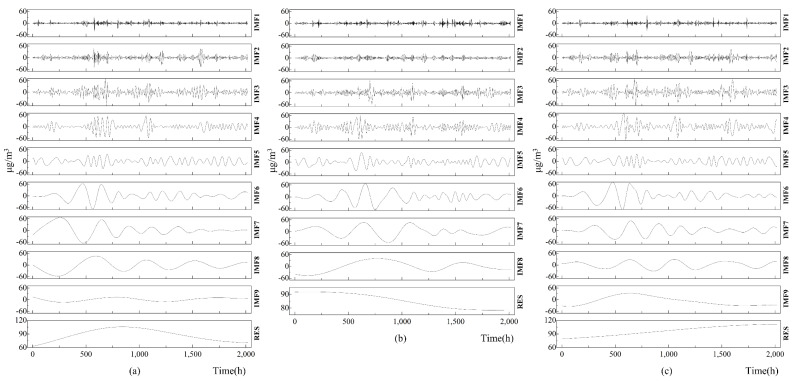
IMFs and residuals of the PM_10_ concentration data series measured at (**a**) Site 1, (**b**) Site 2, and (**c**) Site 3.

To obtain more time scale features from the raw data series, we used EMD to decompose the PM_10_ concentration data measured from the three sites into several prominent IMF components and one residual component, as shown in Figure 4. The figure directly indicates some characteristics of the IMF components. The differences in the number of IMF components at the three measurement sites are apparent. In Figure 3, there are nine independent IMF components at Sites 1 and 2, with eight IMF components from Site 2. The periods of the IMF components exhibited obvious variations. In line with the period lengths, the layout of the IMF components at different sites (Figure 3) was from the shortest to the most extended period. The periods of all IMF components are shown in Table 2.

In Table 2, the longest-period IMF component is more than 1000 h (IMF9 of Site 1, IM8 of Site 2, and IM9 of Site 3), whereas the shortest-period IMF component is only 3.3 h (IMF1 of Sites 1 and 2). More detailed information about the importance of each IMF component relative to all IMF components was obtained by calculating the variance contribution rate, which was calculated as the ratio of the variance of the IMF component to the sum of the variances of all IMF components, as shown in Table 3.

Table 3 shows that the IMF components with a short period, especially the IMF component with a period of less than one day, provided a small contribution to all of the IMF components. Taking IMF1, IMF2, and IMF3 as examples, the variance contribution rates of these three IMF components at Sites 1, 2, and 3 were only 12.4%, 8.34%, and 13.77%, respectively. In the rest of the study, using the Hilbert spectrum, we further analyzed the time–frequency characteristics of the PM_10_ concentration data series.

### 4.3. Characteristics of the Hilbert Spectrum

**Figure 5:**
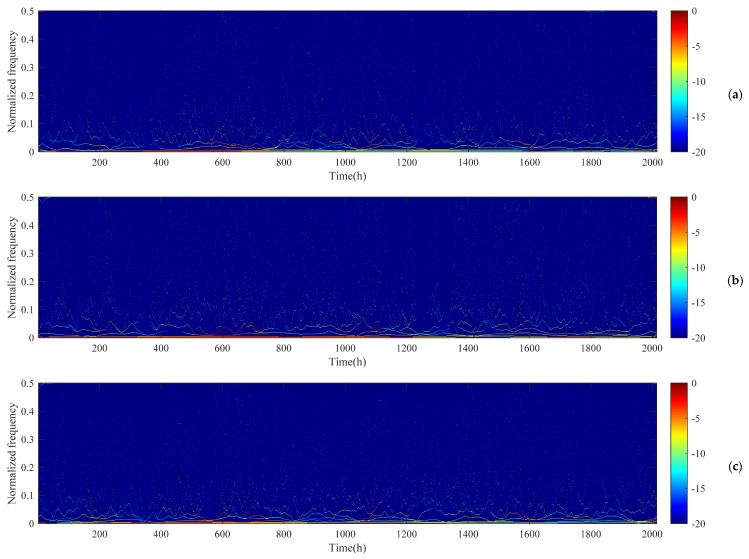
The Hilbert spectrum of the PM_10_ concentration data series measured at (**a**) Site 1, (**b**) Site 2, and (**c**) Site 3.

Applying the HSA technique to the IMF components at different measurement sites, we obtained the Hilbert spectrum of the PM_10_ concentration data series, as shown in Figure 5. The Hilbert spectrum of the PM_10_ concentration data series clearly showed the variation in the amplitude with time (x-axis) and the normalized frequency (y-axis). Here, we found that the variation amplitude of the PM_10_ concentration with time and frequency was discrete, and the range of this variation was mainly concentrated in the low-frequency band (normalized frequency concentrated between 0 and 0.1).

**Figure 6:**
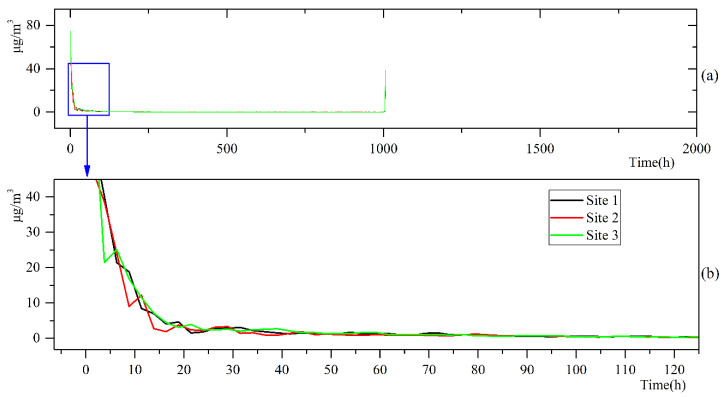
The marginal spectrum of the PM_10_ concentration data series (**a**) and the amplification of the blue box (**b**).

According to Equation (9), the Hilbert spectrum was integrated with the time range in order to obtain the Hilbert marginal spectrum of the PM_10_ concentration data, as shown in Figure 6. We found that the amplitude of the fluctuation in the PM_10_ concentration was mainly concentrated in two frequency bands, corresponding to a short period (about 20 h) and a long period (about 1000 h). We further observed that the fluctuations in the PM_10_ concentration at the three different detection sites were almost the same in the long periods, but showed a distinct difference in the short periods.

Many factors affect the PM_10_ concentration in the port area, and the variations in different elements had a specific time range. Among them, the long-period (approximately 40 days) factors included dry bulk loading and unloading operations (which may last for several weeks). Sorte et al. [16] reported that onshore emission sources, such as trucks, railways, cargo handling equipment, and bulk materials, are the primary contributors of PM_10_, and these emission sources can contribute about 80% of the PM_10_ concentration in a port. Pérez et al. [22] concluded that the PM emitted by port activities contributed about 50–55% of PM_10_ in the harbor. This long-term influencing factor did not significantly contribute to the differences in the PM_10_ concentration data measured at the three sites. In comparison, we focused on the contribution of short-period influencing factors (less than 20 h) to variations in the PM_10_ concentration. As changes in wind speed or direction may only take several hours, we determined that those influencing factors may include wind force, wind direction, temperature, humidity, etc. The HSA of the PM_10_ concentration data obtained from Xinglong Port showed that the short-period influencing factors were the main factors causing the disparity in PM_10_ concentrations at the different measurement sites. However, we did not have detailed log information about the cargo handling and unloading in the port, and could not accurately determine the reliability of those speculations. Furthermore, the period of valid data used in this paper was three months, and it was impossible to judge whether seasonal meteorological characteristics would affect the decomposition of PM_10_ components. In future research, we should combine shipping activity logs, meteorological phenomena, and more extended PM_10_ concentration data in the port in order to explore the correlation among IMF components and various physical phenomena.

Previous studies have proved that obtaining air quality information by deploying a large number of detectors is a prerequisite and necessary condition for air quality assessment and improvement [37,38,39,40,41]. However, strong correlations may exist between the PM_10_ concentration data measured by devices arranged within a specific range [38,41]. Therefore, an unreasonable layout scheme will produce redundant measurement devices, which not only increases the cost of air quality monitoring, but also affects the completeness of air quality information and the accuracy of the air quality assessment. The Xinglong Port case study showed that variability existed in the PM_10_ concentration values at different measurement sites. In Table 1, the maximum difference between the mean values of the PM_10_ concentration data at the three measurement sites was about 7% (the difference between Sites 2 and 3). The difference was insignificant, chiefly caused by short-period factors rather than long-period factors, which will not lead to essential differences in the PM_10_ data series from the different measurement sites. Therefore, through the previous analysis, we can infer that the layout of these three devices in Xinglong Port is unreasonable. On the one hand, the HHT analysis of the PM_10_ concentration data confirms that there are no fundamental differences among the data measured at these three sites, so there is redundancy in the layout of the devices. Also, from the three-month wind direction data, these three measurement sites were all upwind of the port activities. The phenomenon demonstrates that these devices are not placed in suitable measurement sites, and it is hard to accurately measure the impact of port activities on ambient air quality. We think that the corresponding analysis of other ports could be used to determine the reason for the variability in the time–frequency characteristics of the PM_10_ concentration data series, and then to determine whether the layout of the measurement device could be further optimized. That is, the findings here could be adopted as one of the criteria for determining the optimal arrangement of the measurement equipment. Furthermore, to control the PM concentration level around the dry bulk port, the environmental department requires the port authorities to install a windbreak fence around the harbor, increase coverage measures for the cargo yard, and spray during the cargo handling and unloading process. In previous research [36], we found that many factors could influence the effects of these dust suppression measures (such as cargo types, particle size characteristics, and hydrophilicity). However, it is challenging to judge which dust suppression measures are the most necessary in different ports. Combined with the previous analysis, we can determine that the HHT analysis of the PM_10_ concentration data can be used as a useful tool to judge the effects of different measures at the port. In future research, by comparing the HHT analysis of the PM_10_ concentration data in various ports, we could determine whether the physical and chemical characteristics of the cargo have a significant impact on these dust suppression measures. Also, with the HHT analysis and comparison of the PM_10_ concentration data at different scenarios, such as adopting a specific dust suppression measure in different periods, we could reveal whether there are fundamental differences among these various dust suppression measures.

## 5. Conclusions

In this study, we used three online measurement devices deployed in Xinglong Port to obtain a three-month data series, including wind speed, wind direction, and PM_10_ concentration. These time-series data exhibited an apparent difference, of which the differences in wind speed and direction were more noticeable compared with the differences in the mean and variance of the PM_10_ concentration data. The main reasons for the significant differences in wind speeds and forces at the different sites within the port area are the layout of the storage yard and the series of dust suppression measures implemented by the port authority, including the installation of the porous fence and the use of fog cannons. Then, using the Hilbert–Huang transform, we conducted time–frequency characteristic analysis to clarify the reasons for the differences among these PM_10_ concentration data series.

EMD technology is the premise of the Hilbert–Huang transformation; using this technology, we decomposed the PM_10_ concentration data measured at Sites 1, 2, and 3 into nine, eight, and nine IMF components, respectively. The periods of these IMF components exhibited significant differences, with the shortest period being only 3.3 h (IMF1 of Site 1) and the longest period being 1100 h (IMF9 of Site 3). In general, the IMF components with a short period (less than one day) provided a small contribution to all IMF components. Using HSA technology for these IMF components, we found that the variation amplitude in PM_10_ concentration with time and frequency was discrete, and the range of this variation was mainly concentrated in the low-frequency band. By analyzing the Hilbert marginal spectrum, we found that the amplitude of the fluctuation of the PM_10_ concentration was mainly concentrated in two frequency bands, corresponding to a short period (about 20 h) and a long period (about 1000 h). The fluctuations in the PM_10_ concentrations at the three different sites were almost the same in the long period, but exhibit specific differences in the short-term. Thus, we infer that long-period influencing factors (such as cargo loading and unloading operations) determine the PM_10_ concentration levels in the port, and short-period influencing factors (such as wind speed and direction) determine the difference in concentration data at the different sites. As the differences were insignificant (about 7%), we think that the air quality of Xinglong Port can be measured using one instead of three measurement devices, which would reduce the acquisition cost of the PM_10_ concentration data. The method can also be adopted as one of the criteria for determining the optimal arrangement of the measurement equipment.

The limitation of this study is that the length of the analyzed data series was only three months, which may not enable appropriate analysis of the long-term seasonal time–frequency characteristics. Gobbi et al. [19] discovered that ports have a more significant impact on city air quality in spring and summer, and Manoli et al. [20] reported that the median concentrations of PM_10_ in the warm months are higher than those during the cold periods. However, these findings can encourage researchers to pay more attention to the analysis of the time–frequency characteristics of the PM_10_ concentration data.

In summary, we found that the short-period influencing factors are the main factors affecting the difference in the time–frequency characteristics of the PM_10_ concentration data series, and the long-period influencing factors determine the PM_10_ concentration level in the port. Therefore, when formulating PM_10_ emission mitigation strategies, targeted measures must be implemented according to the period of different influencing factors. The results of this study help provide recommendations for port authorities to formulate the optimal layout of measurement devices. Future analysis of the PM_10_ concentration data from different ports (especially over a more extended period) may further reveal other time series characteristics of these data and improve the environmental management capabilities of port authorities.

## Figures and Tables

**Table 1:** Statistical characteristics of PM_10_ concentration data (μg/m^3^).

Site	Maximum	Minimum	Mean	Standard Deviation
1	346.9	18.83	88.77	48.44
2	279.11	18.69	84.61	46.71
3	358.77	18.52	90.91	49.04

**Table 2:** The periods of all intrinsic mode function (IMF) components.

Site	IMF1	IMF2	IMF3	IMF4	IMF5	IMF6	IMF7	IMF8	IMF9
1	3.3	8.3	18.1	35.1	73.3	175.3	268.8	504.0	1008.0
2	3.3	7.1	13.2	27.6	61.1	155.1	403.2	1008.0	-
3	3.4	8.6	17.9	37.0	73.3	149.3	224.0	504.0	1100.0

Notes: ‘-’ means that there is no data here.

**Table 3:** The variance contribution rate of all IMF components (%).

Site	IMF1	IMF2	IMF3	IMF4	IMF5	IMF6	IMF7	IMF8	IMF9
1	0.98	3.33	8.09	9.11	7.51	17.07	27.33	24.09	2.50
2	1.34	1.51	5.49	9.63	8.86	22.13	23.80	27.25	-
3	1.12	3.38	9.27	12.13	7.49	21.67	15.22	10.51	19.23

Notes: ‘-’ means that there is no data here.
